# Research on optimization of basic rail top bending prediction model

**DOI:** 10.1038/s41598-024-60583-9

**Published:** 2024-04-29

**Authors:** Chunjiang Liu, Zhikui Dong, Long Ma, Xinyu Hou, Nanbing Qiao

**Affiliations:** School of Mechanical Engineering, Yanshan University, Hebei Street 438, Qinhuangdao, 066004 Hebei China

**Keywords:** Mechanical engineering, Theory and computation

## Abstract

Since the basic rail of the switch needs to have a certain bending angle when the train changes direction, top bending is an important link in the production process of the basic rail. The three-point pressure top bending method is simple, flexible and widely used. In this study, the traditional three-point pressure bending is optimized, the influence of the pick width in the model is considered, a corresponding rebound model is established, and the model is applied to the pressure bending process of the basic rail. The bilinear strengthening model of the material was used to construct the bending moment expressions at different positions during the top bending process, and the relationship between the load and bending deflection in the elastic stage and elastic-plastic stage was obtained. The final top bending prediction model was obtained by combining the load-deflection model in the bending stage and the rebound stage. The correctness of the theoretical mathematical model was verified by establishing finite element simulations, and the theoretical calculation results were compared with the experimental results. The results showed that the top bend prediction optimization model established in this study had high feasibility and met the machining accuracy requirements.

## Introduction

With the technological change of high-speed railways, trains are developing towards high speed, heavy load and high density, which causes higher requirements for the accuracy and operating life of railway track structure equipment. The basic rail in the turnout is located between the two tongue rails. This connects the normal driving track and the turnout branch track, forming a smooth transition area connecting the two tracks. The contact geometric relationship between the wheel and rail is important for reflecting the dynamic effect of the wheel and rail. As a typical indicator, the basic rail needs to have a certain bending angle for turns as the train is running, and its angle needs to have high accuracy, such that it has high smoothness, high precision and high reliability^1–3^. Therefore, the basic rail needs to be corrected for top bending.

During the three-point bending process, the rail undergoes elastic-plastic deformation. The plastically deformed part cannot return to its original shape, while the elastic part will return to its original state. After the top of the rail bends, its shape changes, and a partial rebound occurs^4^. Since there is always some degree of elastic deformation, the final shape of the object is not the same as the shape from the top bending process. Therefore, the relationship between pressure and deflection during top bending need to be analysed, and the deflection after top bending needs to be predicted.

The mechanical properties of the metal bending process are analysed through the curvature. Stachowicz^5^ investigated the relationship between the bending moment and bending curvature during the bending process of metal pipe fittings, and studied the influence of the bending moment and bending change. Vatter^6^ established a finite element model to simulate the spatial variable curvature forming process and studied the influence of the relevant parameters. Johnson and Yu^7,8^ used linear strengthening and nonlinear power strengthening constitutive models, respectively, to calculate the rebound curvature equation of strip parts during the bending process. Kosel^9^ used a linear elastic hardening rheology model to study the repeated elastic plastic plane pure bending process of a material beam, considered the complete strain history, and described a method for determining the final radius of curvature. Stok and Halilovic^10^ provided an analytical solution for the elastic-plastic bending of rectangular cross-section beams. However, in the actual top bending process, the curvature is very difficult to measure.

Through the analysis of the relationship between the pressure and bending deflection, Cui Fu^11^ proposed the relevant concepts of straightening machinery and straightening theory, and derived a relationship between the load and deflection through the relationship for the curvature and angle. Li Jun et al.^12^ simplified the pressure alignment problem of metals, derived the relationship between the curvature and bending moment, and summarized three calculation methods for the alignment stroke. Song Youshuo^13^ analysed the elastic-plastic mechanics of metal, conducted finite element simulation verification, and determined the residual stress distribution after unloading. Sitar et al.^14^ proposed a numerical calculation method for the elastic-plastic bending and rebound of asymmetric cross-section beams.

Shelest^15^ established an elastic–plastic alternating bending model for the processing of metal strips on roller straightening machines, and calculated the geometric and deformation parameters of the straightening process. Khaleel Ibrahim^16^ employed an optimization method using an objective function to maximize the load and simultaneously constrained the residual internal forces and the complementary strain energy of the steel components to control the residual plastic deformation. Jrad^17^ established the elastic-plastic constitutive equations of the material using a linear hardening model. Nabochenko^18^ proposed the criterion of equivalent bending stiffness for rails. Fallah Nafari^19^ concluded that a mathematical correlation existed among the rail deflection, rail stress, and the load applied to the rail. Zhang^20^ optimized key variables such as span size, straightening position, straightening torque, and straightening stroke.

Most scholars simplify the mechanical model of metal materials into a three-point force model during the three-point bending process; however, the top pick is not just a concentrated force. For this reason, in this study, a corresponding force model was established by considering the influence of the top pick width. The mechanical model was used to calculate the bilinear reinforced constitutive model of the material, and the optimized top bending prediction model was obtained. Finite element simulations were conducted to validate the proposed method against both the model and real experiments, demonstrating the practicality and applicability of the approach^21^. Comparative analysis with other methods confirmed the superiority of the optimized model^22,23^.

## Establishment of the top bending mechanics model

**Figure 1:**
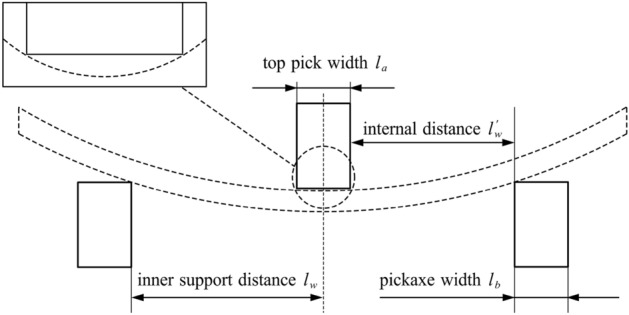
Simplified model of the top bending process.

Compared with traditional three-point top bending, the force exerted by the top pick on the rail is simplified into two concentrated forces in this study. Figure 1 shows the contact relationship between both sides of the top pick and the basic rail for line contact during the top bending process. To simplify the calculation, the basic rail pressure buckling process is simplified to a simple supported beam subject to two concentrated forces. Introducing the top pick and support pick width, the actual top bend support distance is the distance between the inner measurement of the support pick and the centre of the top pick. The inner support distance ${{l}_{w}}$ is the distance between the inner measurements of the two support picks, the outer top distance $l_{w}^{'}$ is the distance between the inner measurement of the support picks and the outside of the top picks, ${{l}_{a}}$ is the width of the top picks, and ${{l}_{b}}$ is the width of the support picks.

**Figure 2:**
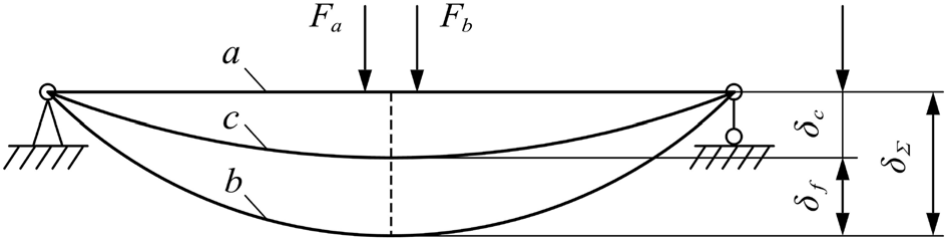
Simplified model of top bending mechanics.

Figure 2 shows a mechanical model of the top bending process. Curve a is the basic rail shape before top bending, curve b is the curved rail shape of the basic rail when top bending reaches the maximum stroke, and curve c is the unloading force of the top pick for the basic rail type after elastic rebound. ${{F}_{a}}$ and ${{F}_{b}}$ are the loads on the basic rail when the top pick is working. They are symmetrically distributed at the centre of the two picks, and the distance is the width of the top pick. ${{\delta }_{g}}$ is the target deflection of top bending, ${{\delta }_{\Sigma }}$ is the maximum top bending stroke in the top bending process, ${{\delta }_{c}}$ is the top bending deflection of the basic rail after top bending is completed, ${{\delta }_{f}}$ is the rebound deflection of the basic rail in the top bending process, and ${{\delta }_{\Sigma }}$ is the top bend stroke. After the rebound is completed and the accuracy requirement of $\left| {{\delta }_{c}}-{{\delta }_{\Sigma }} \right| \le 0.2mm/m$ is met, the top bending process ends.

**Figure 3:**
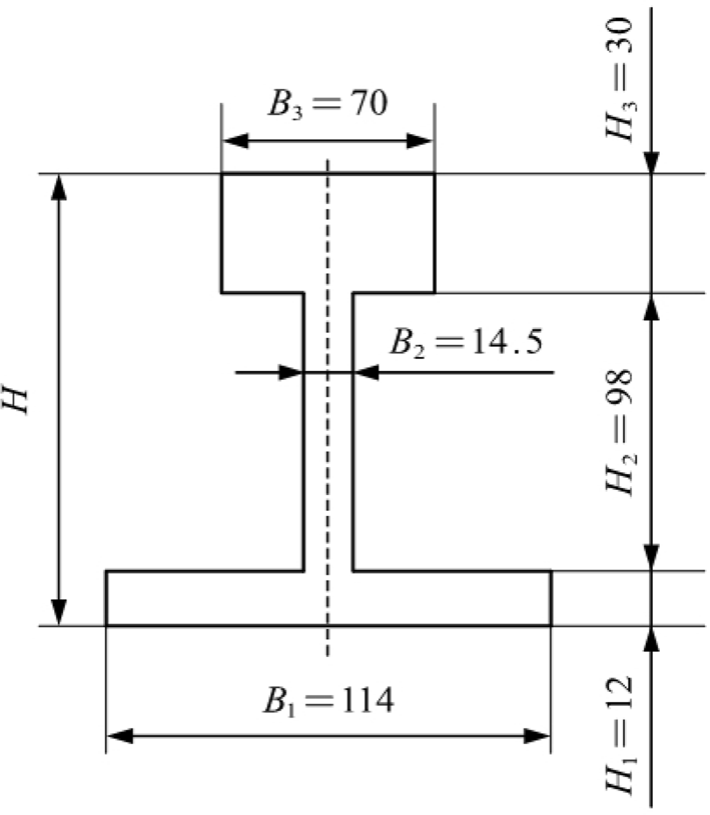
Simplified basic rail section.

Since the basic rail section is relatively complex, the 43 kg/m model basic rail is appropriately simplified according to the method given by Sheng Yanming^24^. The simplified cross-sectional geometric size is basically the same as the original cross-sectional size. By calculating its overall area, the horizontal moment of inertia, centroid position and other parameters, the error between the simplified parameters and the original parameters is low, and the formula based on this can be easily derived. The simplified shape and size information is shown in Fig. 3.

**Figure 4:**
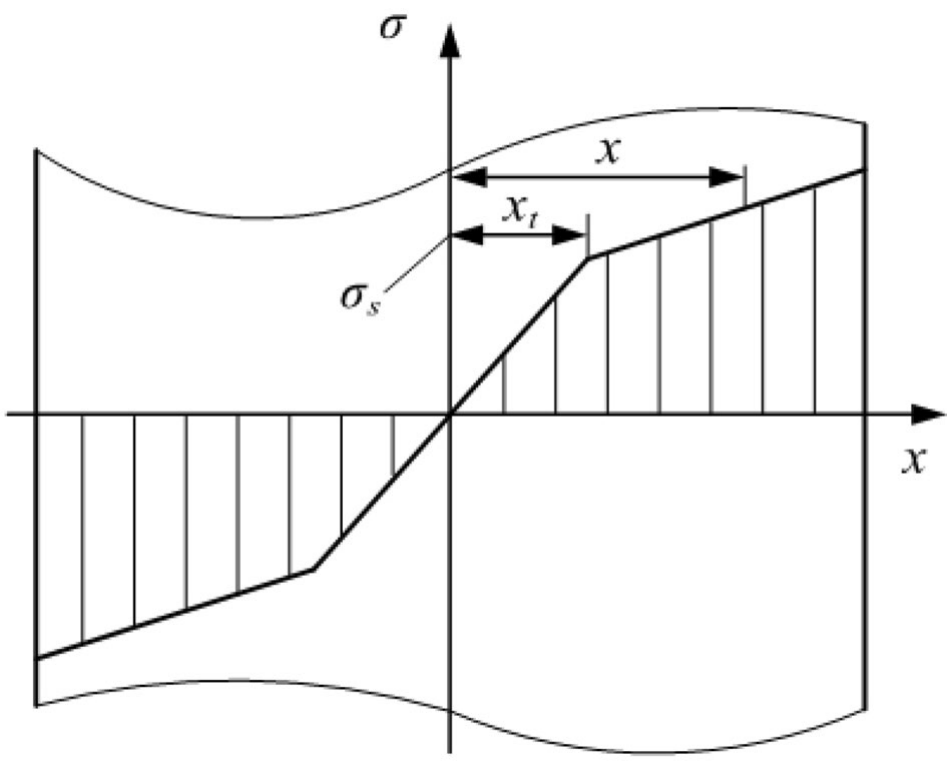
Stress distribution of the basic rail section.

To simplify the calculation, the bilinear strengthening model of the material is used to calculate the bending moment, as shown in Fig. 4; this is the distribution of the cross-sectional stress.

**Figure 5:**
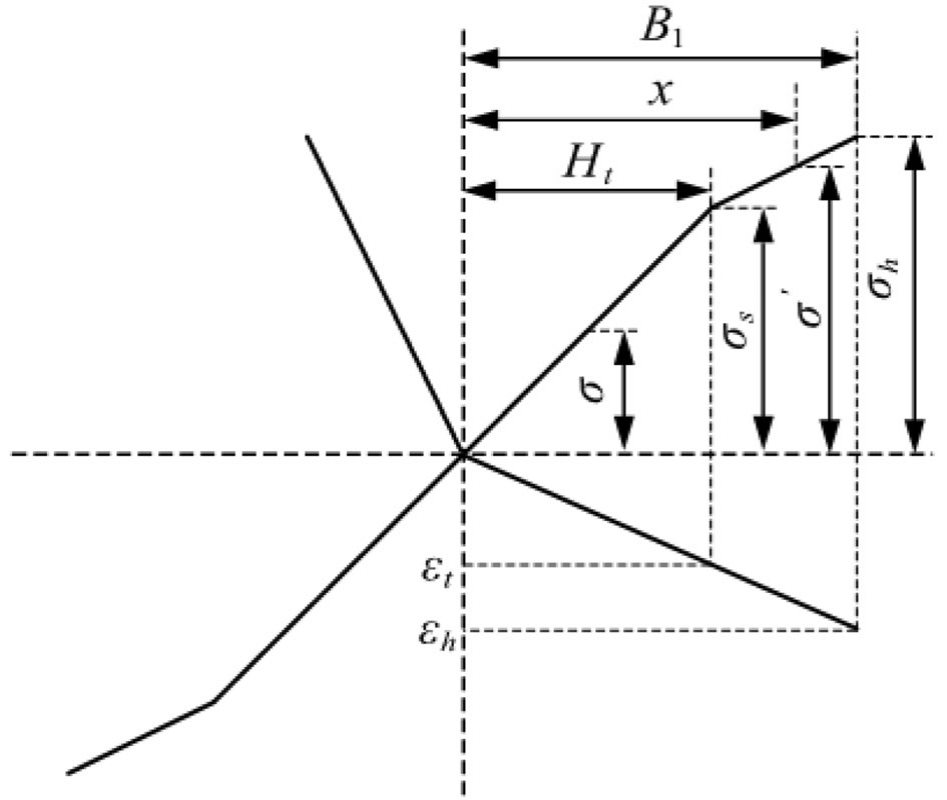
Stress and strain in elastic-plastic bending.

Figure 5 shows the stress strain during elastic-plastic bending, where ${{H}_{t}}$ is half the thickness of the elastic deformation layer. The stress magnitude in the elastic deformation layer is as shown in Eq. (1):

1 $$\begin{aligned} \sigma =\frac{x{{\sigma }_{s}}}{{{H}_{t}}} \end{aligned}$$

When the layer is plastically deformed, Eq. (2) is determined using the proportional relationship:

2 $$\begin{aligned} \left\{ \begin{aligned}&\frac{{{\sigma }^{'}}-{{\sigma }_{s}}}{x-{{H}_{t}}}=\frac{{{\sigma }_{h}}-{{\sigma }_{s}}}{{{B}_{1}}-{{H}_{t}}} \\&\quad {{\sigma }_{h}}-{{\sigma }_{s}}={{E}_{t}}({{\varepsilon }_{h}}-{{\varepsilon }_{t}}) \\ \end{aligned} \right. \end{aligned}$$

Through the above equation, Eq. (3) can be obtained:

3 $$\begin{aligned} {{\sigma }^{'}}-{{\sigma }_{s}}=\frac{{{E}_{t}}{{\sigma }_{s}}(x-{{H}_{t}})}{E{{H}_{t}}} \end{aligned}$$

From the linear strengthening coefficient $\lambda =\frac{{{E}_{t}}}{E}$, we obtain Eq. (4):

4 $$\begin{aligned} {{\sigma }^{'}}=\left[ 1+\lambda (\frac{x}{{{H}_{t}}}-1) \right] {{\sigma }_{s}} \end{aligned}$$

**Figure 6:**
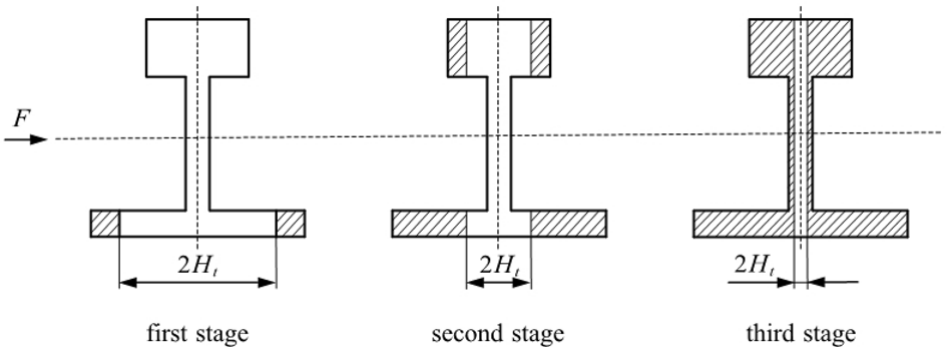
Three stages of the elastic-plastic deformation of the basic rail.

After the cross-section is simplified, the basic rail can be divided into three parts: the rail head, rail waist and rail bottom. Analysis of the top bending process of the basic rail shows that it can be divided into three stages when elastic-plastic deformation occurs, as shown in Fig. 6. Whenever the plastic deformation reaches a sudden change in the section, the elastic-plastic calculation formula changes. According to the plastic deformation reaching different parts of the basic rail section, the bending moment is analysed and calculated in each stage.

1. Elastic limit bending moment
  When the basic rail bends laterally, elastic deformation initially occurs. At this time, the stress-strain relationship conforms to Hooke’s law. According to Fig. 5, the elastic limit bending moment of the basic rail during the bending process is given by Eq. (5):
  5 $$\begin{aligned} {{M}_{t}}=2\int _{0}^{\frac{B1}{2}}{\sigma {{H}_{1}}xdx}+2\int _{0}^{\frac{B2}{2}}{\sigma {{H}_{2}}xdx}+2\int _{0}^{\frac{{{B}_{3}}}{2}}{\sigma {{H}_{3}}xdx} \end{aligned}$$
  From the formula, $\sigma =\frac{2x}{{{B}_{1}}}{{\sigma }_{s}}$ is substituted into the above formula, and Eq. (6) is obtained:
  6 $$\begin{aligned} {{M}_{t}}=\frac{{{H}_{1}}B_{1}^{3}+{{H}_{2}}B_{2}^{3}+{{H}_{3}}B_{3}^{3}}{6{{B}_{1}}}{{\sigma }_{s}} \end{aligned}$$
  According to the calculation formula $W=\frac{M}{\sigma }$, the bending section coefficient of the basic rail is obtained as shown in Eq. (7):
  7 $$\begin{aligned} W=\frac{{{H}_{1}}B_{1}^{3}+{{H}_{2}}B_{2}^{3}+{{H}_{3}}B_{3}^{3}}{6{{B}_{1}}} \end{aligned}$$
2. Elastoplastic bending moment
  Figure 6 shows that the elastic-plastic deformation of the basic rail during the top bending process can be divided into three stages. In the first stage, plastic deformation occurs only at the rail bottom. In the second stage, plastic deformation occurs at both the rail bottom and the rail. In the third stage, the plastic deformation occurs at the rail bottom, rail head and rail waist. The elastic zone ratio $\xi$ is set as the ratio of the elastic zone length to the total length, that is, $\xi ={{H}_{t}}/H$. To prevent the basic rail from the plastic hinges due to deformation, the following is defined: ${{\xi }_{\min }}=0.2$.

In the first stage, plastic deformation occurs only at the bottom of the rail.

The bending moment expression is established as shown in Eq. (8):

8 $$\begin{aligned} {{M}_{1}}=2\int _{0}^{\frac{{{H}_{t}}}{2}}{\sigma {{H}_{1}}xdx}+2\int _{\frac{{{H}_{t}}}{2}}^{\frac{{{B}_{1}}}{2}}{{{\sigma }^{'}}{{H}_{1}}xdx}+2\int _{0}^{\frac{{{B}_{2}}}{2}}{\sigma {{H}_{2}}xdx}+2\int _{0}^{\frac{{{B}_{3}}}{2}}{\sigma {{H}_{3}}xdx} \end{aligned}$$

The formula for sorting is shown in Eq. (9):

9 $$\begin{aligned} {{M}_{1}}=\left\{ \frac{{{H}_{1}}B_{1}^{2}(\lambda -1)}{12}{{\xi }^{2}}+\left[ \frac{{{H}_{1}}B_{1}^{2}(\lambda -1)}{6}+W \right] \frac{1}{\xi }+\frac{{{H}_{1}}B_{1}^{2}(1-\lambda )}{4} \right\} {{\sigma }_{s}} \end{aligned}$$

In the second stage, plastic deformation occurs at the rail bottom and rail head.

The bending moment expression is established as shown in Eq. (10):

10 $$\begin{aligned} {{M}_{2}}=2\int _{0}^{\frac{{{B}_{2}}}{2}}{\sigma {{H}_{2}}xdx}+2\int _{0}^{\frac{{{H}_{t}}}{2}}{\sigma {{H}_{1}}xdx}+2\int _{\frac{{{H}_{t}}}{2}}^{\frac{{{B}_{1}}}{2}}{{{\sigma }^{'}}{{H}_{1}}xdx}+2\int _{0}^{\frac{{{B}_{3}}}{2}}{\sigma {{H}_{3}}xdx}+2\int _{\frac{{{H}_{t}}}{2}}^{\frac{{{B}_{3}}}{2}}{{{\sigma }^{'}}{{H}_{3}}xdx} \end{aligned}$$

The formula for sorting is shown in Eq. (11):

11 $$\begin{aligned} {{M}_{2}}=\left\{ \frac{\left( {{H}_{1}}+{{H}_{3}} \right) B_{1}^{2}(\lambda -1)}{12}{{\xi }^{2}}+\left[ \frac{\left( {{H}_{1}}B_{1}^{3}+{{H}_{3}}B_{3}^{3} \right) (\lambda -1)}{6{{B}_{1}}}+W \right] \frac{1}{\xi }+\frac{\left( {{H}_{1}}B_{1}^{2}+{{H}_{3}}B_{3}^{2} \right) (1-\lambda )}{4} \right\} {{\sigma }_{s}} \end{aligned}$$

In the third stage, plastic deformation occurs at the rail bottom, rail head and rail waist.

The bending moment expression is established as shown in Eq. (12):

12 $$\begin{aligned} {{M}_{3}}= & \,2\int _{0}^{\frac{{{H}_{t}}}{2}}{\sigma {{H}_{1}}xdx}+2\int _{\frac{{{H}_{t}}}{2}}^{\frac{{{B}_{1}}}{2}}{{{\sigma }^{'}}{{H}_{1}}xdx}+2\int _{0}^{\frac{{{H}_{t}}}{2}}{\sigma {{H}_{2}}xdx}+2\int _{\frac{{{H}_{t}}}{2}}^{\frac{{{B}_{2}}}{2}}{{{\sigma }^{'}}{{H}_{2}}xdx}\nonumber \\{} & +2\int _{0}^{\frac{{{H}_{t}}}{2}}{\sigma {{H}_{3}}xdx}+2\int _{\frac{{{H}_{t}}}{2}}^{\frac{{{B}_{3}}}{2}}{{{\sigma }^{'}}{{H}_{3}}xdx} \end{aligned}$$

The formula for sorting is shown in Eq. (13):

13 $$\begin{aligned} {{M}_{3}}=\left\{ \frac{\left( {{H}_{1}}+{{H}_{2}}+{{H}_{3}} \right) B_{1}^{2}(\lambda -1)}{12}{{\xi }^{2}}+\lambda W\frac{1}{\xi }+\frac{\left( {{H}_{1}}B_{1}^{2}+{{H}_{2}}B_{2}^{2}+{{H}_{3}}B_{3}^{2} \right) (1-\lambda )}{4} \right\} {{\sigma }_{s}} \end{aligned}$$

When plastic deformation occurs during the top bending process, the corresponding bending moment formulas at different positions are shown in Eq. (14):

14 $$\begin{aligned} {{M}_{\lambda }}=\left( a+b{{\xi }^{2}}+\frac{c}{\xi } \right) {{\sigma }_{s}}=\left( a+b{{\xi }^{2}}+\frac{c}{\xi } \right) \frac{{{M}_{t}}}{W} \end{aligned}$$

The values of a, b, and c are obtained according to the stage of plastic deformation during the top bending process.

## Establishment of the top bend deflection model

**Figure 7:**
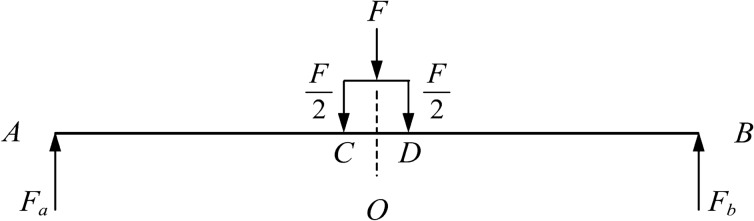
Force analysis.

The stress analysis of the top bending process of the basic rail is shown in Fig. 7.

**Figure 8:**
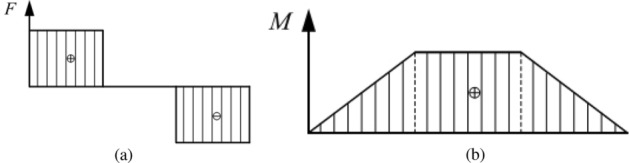
Shear force diagram and bending moment diagram.

The results from the force and bending moment at different positions are shown in Eqs. (15)–(20), and the shear force diagram and bending moment diagram are shown in Fig. 8.

The equations in the AC segment are defined as follows:

15 $$\begin{aligned} {{F}_{x}}=  {{F}_{A}}=\frac{1}{2}F \end{aligned}$$

16 $$\begin{aligned} {{M}_{x}}= {{F}_{A}}x=\frac{1}{2}Fx \end{aligned}$$

The equations in the CD segment are defined as follows:

17 $$\begin{aligned} {{F}_{x}}= {{F}_{A}}-\frac{1}{2}F=0 \end{aligned}$$

18 $$\begin{aligned} {{M}_{x}}= {{F}_{A}}x-\frac{1}{2}F\left( x-l_{w}^{'} \right) =\frac{1}{2}Fl_{w}^{'} \end{aligned}$$

The equations in the BD segment are defined as follows:

19 $$\begin{aligned} {{F}_{x}}= -{{F}_{B}}=-\frac{1}{2}F \end{aligned}$$

20 $$\begin{aligned} {{M}_{x}}= {{F}_{B}}\left( 2{{l}_{w}}-x \right) =\frac{1}{2}F\left( 2{{l}_{w}}-x \right) \end{aligned}$$

According to the internal and external moment balance, the equilibrium relationship in the elastic-plastic stage can be obtained as shown in Eqs. 21 and 22.

The equations in the AC segment are defined as follows:

21 $$\begin{aligned} \frac{1}{2}Fx=\left( a+b{{\xi }^{2}}+\frac{c}{\xi } \right) \frac{{{M}_{t}}}{W} \end{aligned}$$

The equations in the CO segment are defined as follows:

22 $$\begin{aligned} \frac{1}{2}Fl_{w}^{'}=\left( a+b{{\xi }^{2}}+\frac{c}{\xi } \right) \frac{{{M}_{t}}}{W} \end{aligned}$$

The top bending of the basic rail is usually carried out by a press. The top bending force is macroscopically expressed as a concentrated force. Through the contact between the top pick and the basic rail, a concentrated force is divided into two concentrated forces of equal size and symmetrical distribution. To ensure that the deformation of the basic rail will not cause defects, the force *F* needs to be limited, and its variation range is shown in Eq. (23):

23 $$\begin{aligned} {{F}_{t}}\le F\le {{F}_{\max }} \end{aligned}$$

In the above formula, ${{F}_{t}}$ is defined as follows:${{F}_{t}}=\frac{2{{M}_{t}}}{l_{w}^{'}}$

By using the above relationships, Eqs. (24) and (25) can be obtained.

The equations in the AC segment are defined as follows:

24 $$\begin{aligned} x=\frac{l_{w}^{'}}{\alpha W}\left( a+b{{\xi }^{2}}+\frac{c}{\xi } \right) \end{aligned}$$

The equations in the CO segment are defined as follows:

25 $$\begin{aligned} \alpha W=\left( a+b{{\xi }^{2}}+\frac{c}{\xi } \right) \end{aligned}$$

**Figure 9:**
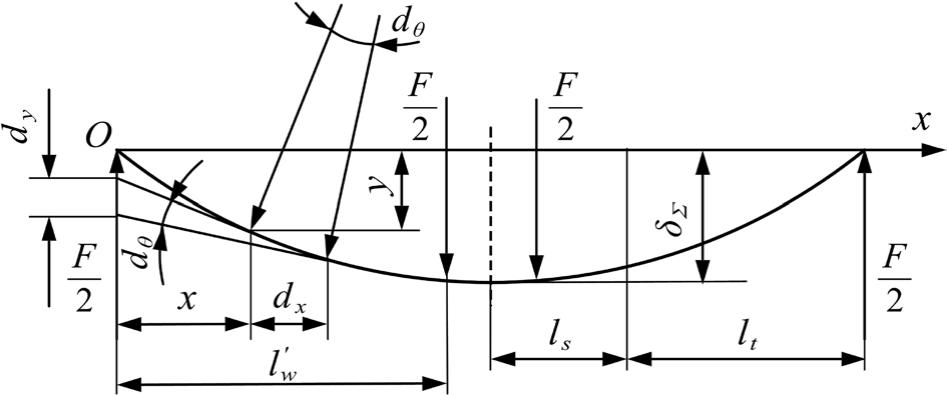
Schematic diagram of the top bending process.

Figure 9 shows a schematic diagram of the top bending process of the basic rail. ${{l}_{t}}$ is the length of the elastic region of one side of the basic rail, and ${{l}_{s}}$ is the length of the elastic-plastic region of one side. According to the shear force diagram and bending moment diagram, the force and bending moment on the basic rail are symmetrical; thus, in this study, the left side of the symmetry axis is only examined.

Since the bending inclination caused by the top bend is not large, the short segment length ${{\text {d}}_{x}}$ of the horizontal axis can be regarded as the short segment arc length ${{\text {d}}_{s}}$ in the deflection curve. Assuming that the top curvature rate at x is ${{C}_{x}}$ and the corresponding radius of curvature is ${{\rho }_{x}}$, the change in rotation angle can be calculated as ${{d}_{\theta }}={{d}_{x}}/{{\rho }_{x}}={{C}_{x}}{{d}_{x}}$. Due to the small deformation relationship, the deflection change at x can be calculated simply as ${{d}_{y}}=x{{d}_{\theta }}=x{{C}_{x}}{{d}_{x}}$. Then, the calculation formula for the reverse bending deflection corresponding to the midpoint is shown in Eq. (26):

26 $$\begin{aligned} {{\delta }_{\Sigma }}=\int _{0}^{\delta }{{{d}_{y}}}=\int _{0}^{l_{w}^{'}}{x{{C}_{x}}}{{d}_{x}} \end{aligned}$$

The integral of the above piecewise formula is calculated as follows: $0\sim {{l}_{t}}$ and ${{l}_{t}}\sim l_{w}^{'}$. There are elastic deformation areas at both ends of the basic rail. When the force $\frac{F}{2}$ is constant, the elastic area length ${{l}_{t}}$ is also constant. Therefore, in the elastic region, the ${{C}_{x}}$ function is a linear function, as shown in Eq. (27).

27 $$\begin{aligned} {{C}_{x}}=\frac{{{M}_{x}}}{EI}=\frac{Fx}{2EI} \end{aligned}$$

The formula is substituted into the first part of the calculation formula, as shown in Eq. (28):

28 $$\begin{aligned} {{\delta }_{{{\Sigma }_{1}}}}=\int _{0}^{{{l}_{t}}}{\frac{F{{x}^{2}}}{2EI}}{{d}_{x}}+\int _{{{l}_{t}}}^{l_{w}^{'}}{x{{C}_{x}}dx} =\frac{Fl_{t}^{3}}{6EI}+\int _{{{l}_{t}}}^{l_{w}^{'}}{x{{C}_{x}}dx} \end{aligned}$$

The calculation formula for the second part is shown in Eq. (29):

29 $$\begin{aligned} {{\delta }_{{{\Sigma }_{2}}}}=\int _{l_{w}^{'}}^{l_{w}^{'}+\frac{{{l}_{a}}}{2}}{x{{C}_{x}}dx} \end{aligned}$$

## Establishment of the top curvature model

**Figure 10:**
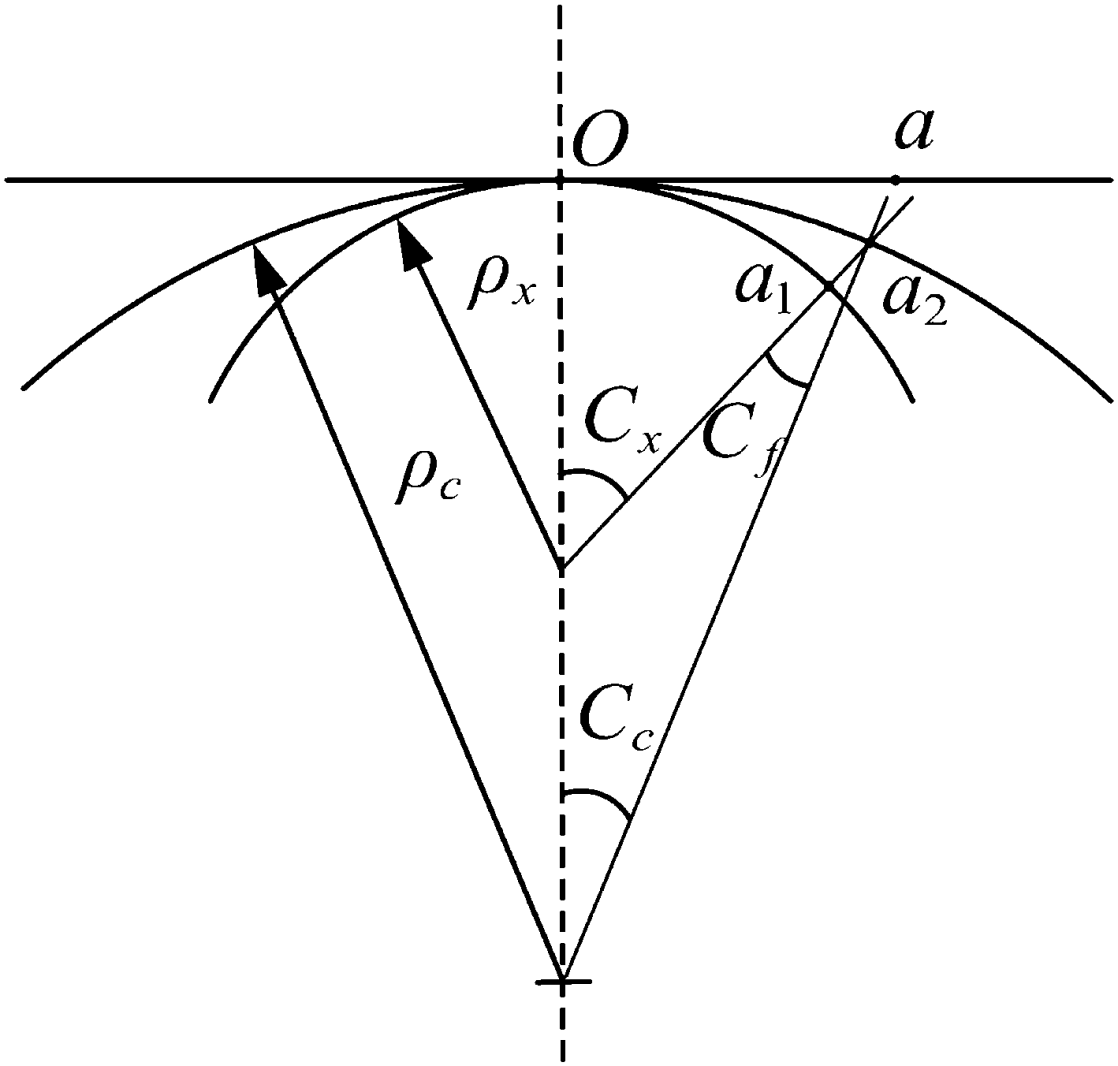
Change in curvature during bending.

The stress state of the basic rail during the top bending process is shown in Fig. 10. In the geometric analysis, the curvature and deformation of the bending are considered from the micro-line segments. The original state of the basic rail before the top bending is straight. The unit length is defined as follows: $Oa=1$. When bending occurs, the corresponding arc centre angle is *C*, the arc length is $\overset{\frown }{Oa}=1$, and the radius of curvature is $\rho$. The correspondence between them is shown in Eq. (30):

30 $$\begin{aligned} C=\frac{\overset{\frown }{Oa}}{\rho }=\frac{1}{\rho } \end{aligned}$$

The arc centre angle *C* is the same as the curvature of the arc, but the unit dimension is changed from *rad* to ${{m}^{-1}}$; thus, the curvature can be replaced by the arc centre angle *C*. The state corresponding to the total stroke of the top bending process is bent from a straight line *Oa* to a curve $\overset{\frown }{O{{a}_{1}}}$. At this time, the top bending rate is ${{C}_{x}}$, and the radius of curvature is ${{\rho }_{x}}$. The corresponding relationship is shown in Eq. (31):

31 $$\begin{aligned} {{C}_{x}}=\frac{1}{{{\rho }_{x}}} \end{aligned}$$

The state corresponding to the rebound stage of the top bending process rebounds from curve $\overset{\frown }{O{{a}_{1}}}$ to curve $\overset{\frown }{O{{a}_{2}}}$. At this time, the top bending rate is ${{C}_{c}}$, the radius of curvature is ${{\rho }_{c}}$, and the rebound curvature is ${{C}_{f}}$. The corresponding relationship is shown in Eq. (32):

32 $$\begin{aligned} {{C}_{c}}=\frac{1}{{{\rho }_{c}}} \end{aligned}$$

The elastic limit curvature is set to ${{C}_{t}}$, the outermost fibre of the basic rail just reaches the elastic limit stage, and its calculation expression is shown in Eq. (33):

33 $$\begin{aligned} {{C}_{t}}=\frac{2{{\varepsilon }_{t}}}{{{H}_{{}}}} \end{aligned}$$

The curvature ratio is the ratio of the curvature to the elastic limit of curvature, represented by *A*, where the total bending curvature ratio is given by Eq. (34):

34 $$\begin{aligned} {{A}_{x}}=\frac{{{C}_{x}}}{{{C}_{t}}} \end{aligned}$$

According to the principle of plane section, ${{C}_{x}}=\frac{2{{\varepsilon }_{t}}}{{{H}_{t}}}$ can be obtained; by using this relationship, we can obtain Eq. (35):

35 $$\begin{aligned} {{A}_{x}}=\frac{{{C}_{x}}}{{{C}_{t}}}=\frac{2{{\varepsilon }_{t}}H}{2{{\varepsilon }_{t}}{{H}_{t}}}=\frac{H}{{{H}_{t}}} \end{aligned}$$

By setting the bomb area ratio $\xi$, we can obtain Eq. (36):

36 $$\begin{aligned} {{C}_{x}}=\frac{{{C}_{t}}}{\xi } \end{aligned}$$

## Establishment of the top bend deflection model

**Figure 11:**
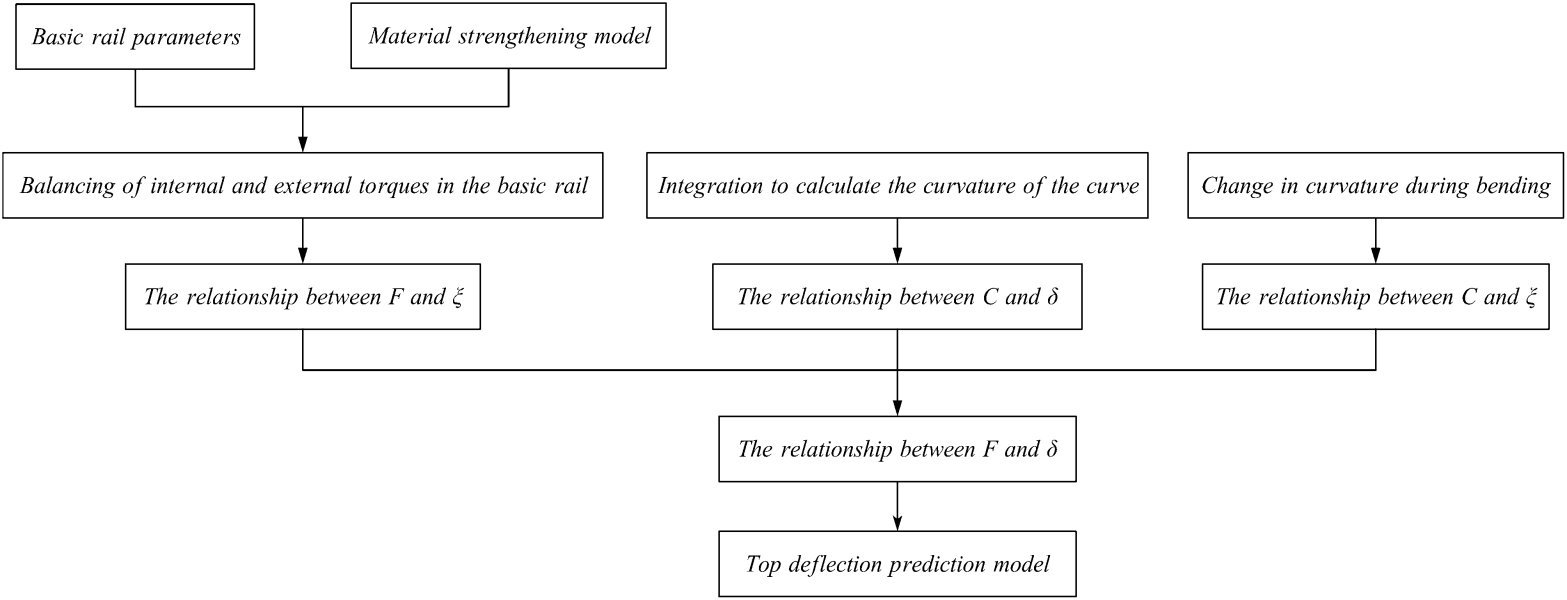
Calculation flowchart.

Based on the analysis presented earlier, a flowchart is constructed, as shown in Fig. 11, and the relationship are shown in Eqs. 37 and 38. The trapezoidal method is used to calculate the integral. The total deflection calculation formula for the top bending process is ${{\delta }_{\Sigma }}={{\delta }_{{{\Sigma }_{1}}}}+{{\delta }_{{{\Sigma }_{2}}}}$. The corresponding relationship between *F* and ${{\delta }_{\Sigma }}$ can be obtained. The load-deflection curves of the different types of basic rails and the load-deflection relationships during the top bending process with different top pick widths are plotted.

37 $$\begin{aligned}{} & {} \left\{ \begin{aligned}&{{F}_{1}}=\alpha \frac{2{{M}_{t}}}{l_{w}^{'}}\left( \alpha =1\rightarrow \frac{a+b\xi _{\min }^{2}+\frac{c}{{{\xi }_{\min }}}}{W} \right) \\&{{\delta }_{{{\Sigma }_{1}}}}=\frac{Fl_{t}^{3}}{6EI}+\int _{{{l}_{t}}}^{l_{w}^{'}}{x{{C}_{x}}dx} \\&{{C}_{x}}=\frac{{{C}_{t}}}{\xi } \\&{{x}_{1}}=\frac{l_{w}^{'}}{\alpha W}\left( a+b{{\xi }^{2}}+\frac{c}{\xi } \right) \\ \end{aligned} \right. \end{aligned}$$

38 $$\begin{aligned}{} & {} \left\{ \begin{aligned}&{{F}_{2}}={{F}_{1}} \\&{{\delta }_{{{\Sigma }_{2}}}}=\int _{l_{w}^{'}}^{l_{w}^{'}+\frac{{{l}_{a}}}{2}}{x{{C}_{x}}dx} \\&{{C}_{x}}=\frac{{{C}_{t}}}{\xi } \\&\alpha W=\left( a+b{{\xi }^{2}}+\frac{c}{\xi } \right) \\ \end{aligned} \right. \end{aligned}$$

## Establishment of the top curvature model

**Figure 12:**
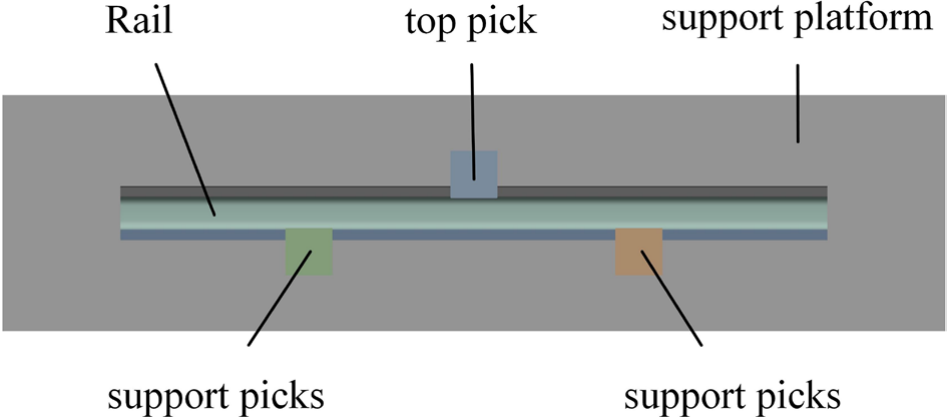
Finite element simulation model of top bending.

According to the actual working status, a simplified finite element simulation model is constructed^25^, as shown in Fig. 12.

The model mainly includes a support platform, experimental rails, a top pick that exerts pressure on the rails, and two support picks that play a supporting role. On one side of the rail, two picks are set as the fulcrum for bending the rail according to the distance between the picks, as shown in Table 1, and picks of different widths are set on the other side to bend the rail. The bending parameters of the curved rails used are shown in Table 1.

**Table 1:** Finite element simulation parameters of the top-bending rails.

Rail material	Top pick downwards distance (mm)	Top pick width (mm)	Pick distance (mm)
U71Mn	8	100–300	600–1000

A finite element simulation model was established based on the actual top bending process. During the top bending process, the deformation of the support platform, top pick, and support pick was negligible; thus, they were set as analytical rigid bodies. The friction between each component during the top bending process had a small impact on the top bending, and the contact between each component was set using the default friction contact. At the same time, the simulation was performed in three analysis steps. The first analysis step applied displacement. The displacement was the value of the top bending stroke calculated theoretically to achieve the reverse bending of the rail. In the second analysis step, pressure maintenance is performed. In the third analysis step, the rail rebounds as the pressure is unloaded step by step.

**Figure 13:**
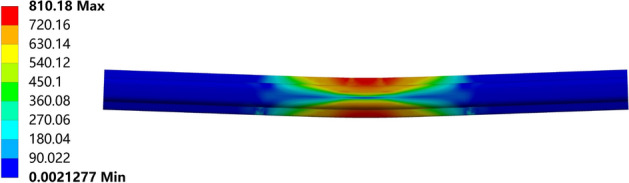
After top bending.

**Figure 14:**
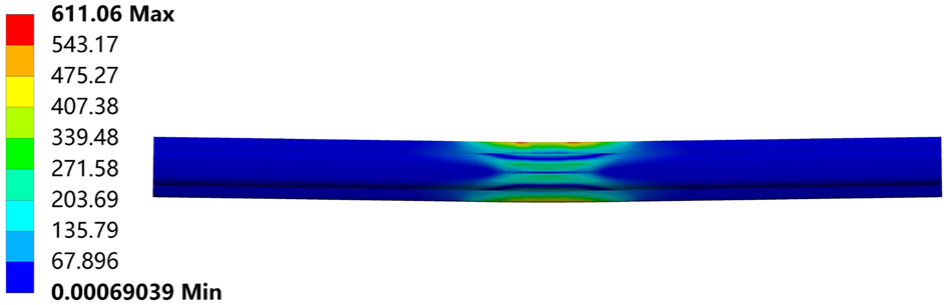
After rebound.

By changing the fixed top die width in the existing model, models with different widths of support dies and different distances between support dies are introduced. The influence of different top die widths on the bending effect is verified through simulation. The simulated stress cloud diagrams of the rail after bending and rebound are shown in Figs. 13 and 14, respectively.

**Figure 15:**
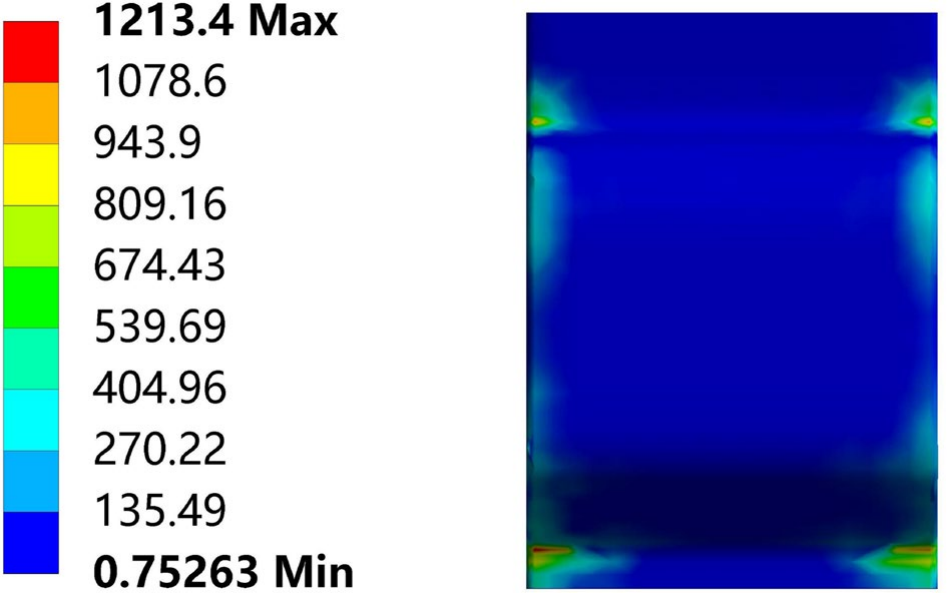
Stress diagram of the top pick.

During the top bending simulation process, the stress state of the top pick was observed, as shown in Fig. 15; these results were consistent with the two concentrated forces proposed by this study, where the top pick provided two concentrated forces.

**Figure 16:**
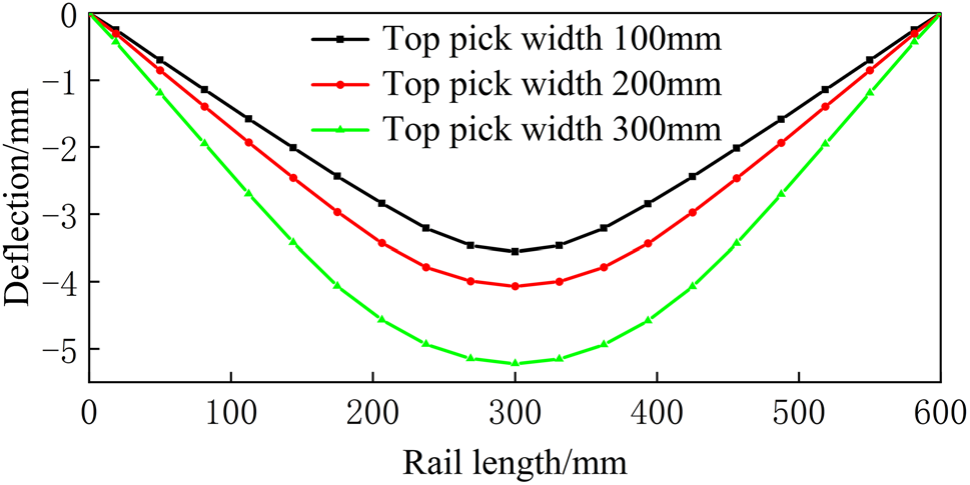
Spread of 600 mm.

**Figure 17:**
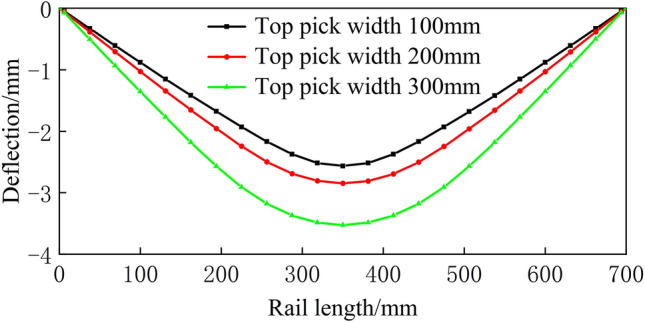
Spread of 700 mm.

**Figure 18:**
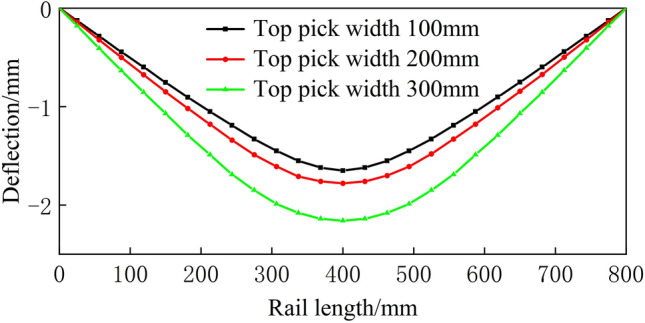
Spread of 800 mm.

**Figure 19:**
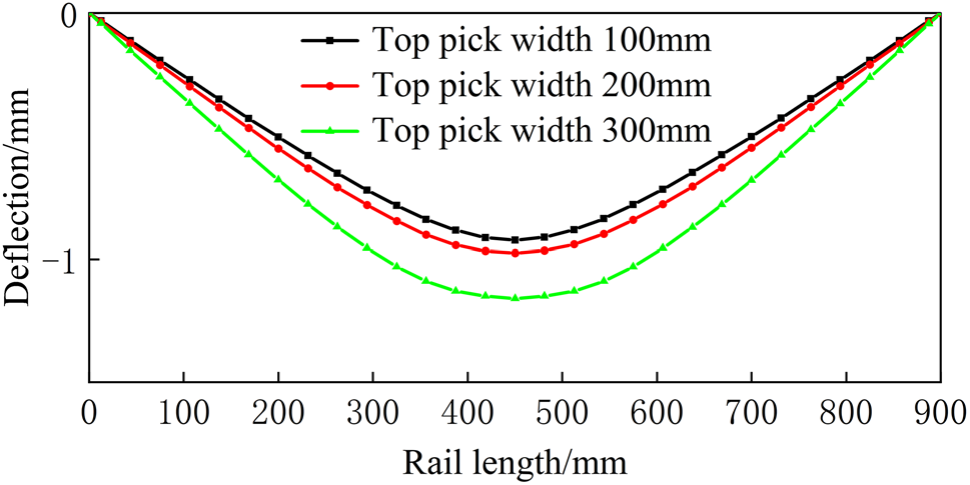
Spread of 900 mm.

**Figure 20:**
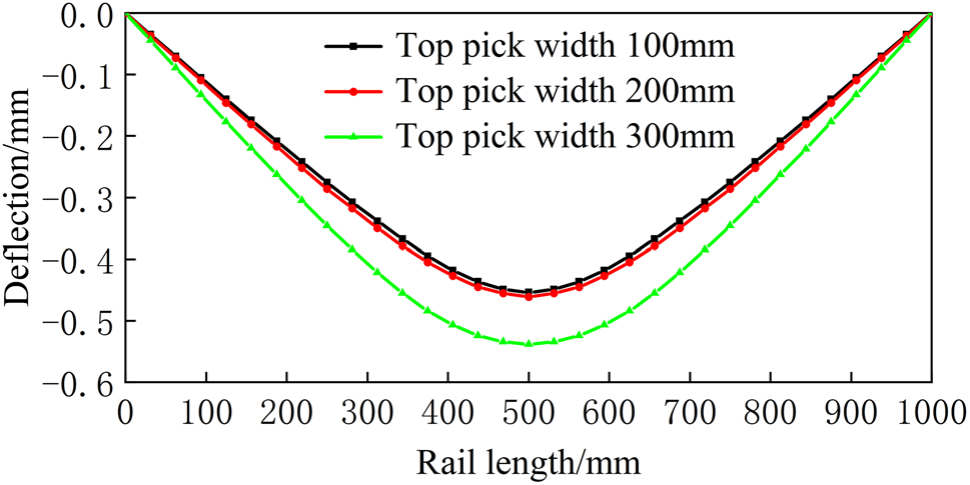
Spread of 1000 mm.

The finite element top bending simulation of the rail is carried out according to the parameters provided in Table 1. The working edge deflection curves under different working conditions after top bending are shown in Figs. 16, 17, 18, 19 and 20.

From Figs. 16, 17, 18, 19 and 20, a significant difference in deflection is observed between the top die widths of 300 mm and 100 mm. These results indicate that the top die width has a significant impact on the residual deflection after bending. Additionally, some errors occur under different top die widths. To further reduce these errors, the influence of the top die width on the prediction model for the post-bending deflection need to be considered.

**Figure 21:**
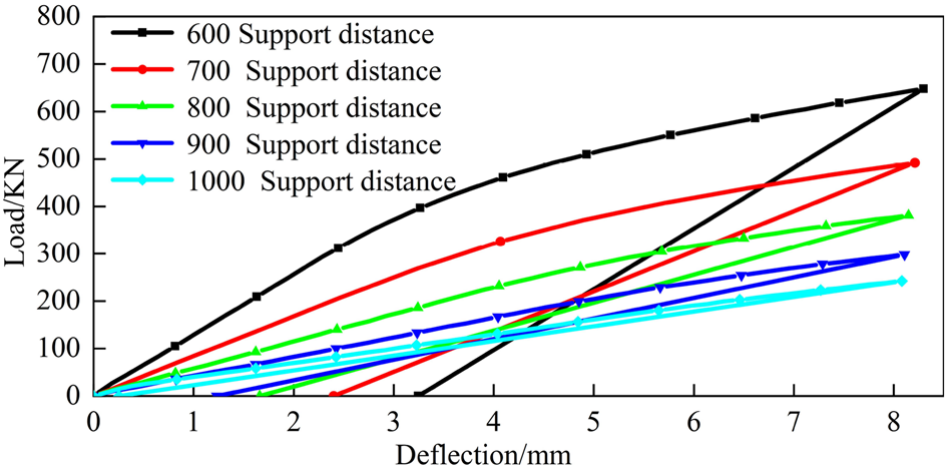
Top pick width of 100 mm.

**Figure 22:**
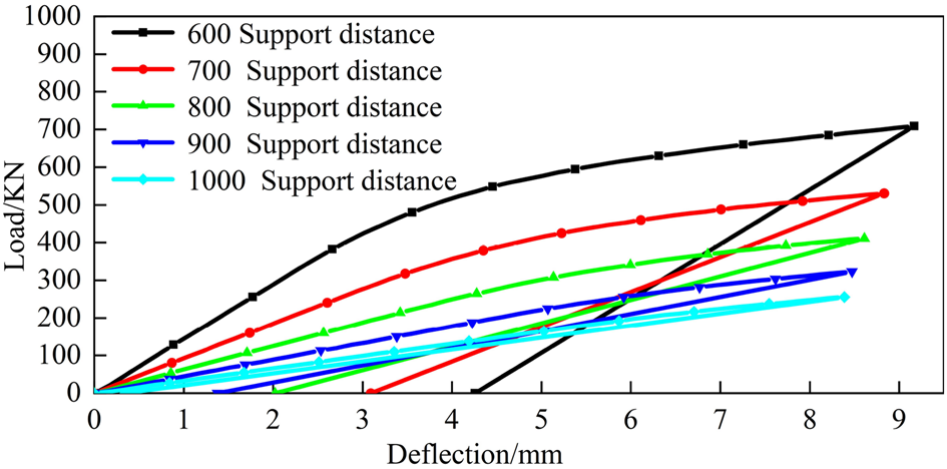
Top pick width of 200 mm.

**Figure 23:**
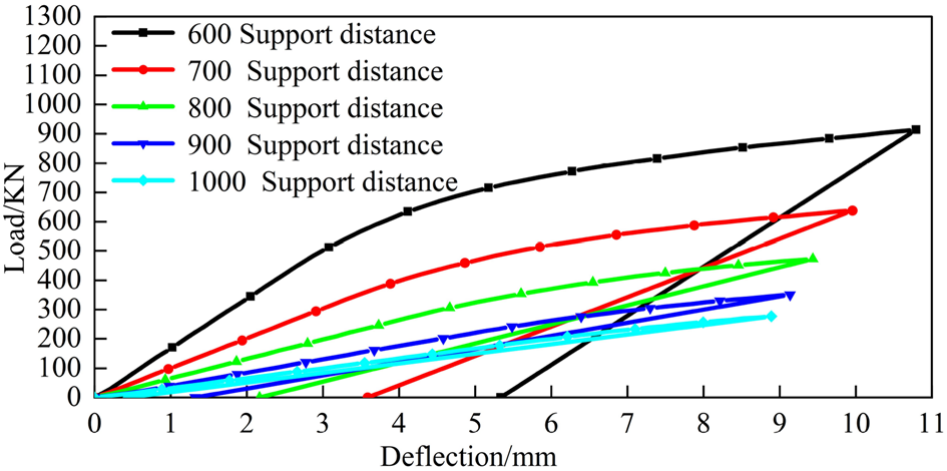
Top pick width of 300 mm.

The top pick stroke is set to 8 mm, the finite element simulation data are obtained, the corresponding load-deflection model is established under different top pick widths and different support distances, and the relationship between the top-bending load and the top-bending stroke is analysed. Figures 21, 22 and  23 show the load-deflection models under different top pick widths and different support distances.

From Figs. 21, 22 and 23, the forms of the load-deflection curves under different jack widths and different support distances are the same; specifically, they are divided into the elastic loading stage, elastic-plastic loading stage and linear rebound stage. These results are consistent with the theoretically calculated load-deflection model.

Furthermore, as the top die width increases under fixed bending stroke conditions, the rail deflection gradually increases along with the load. Consequently, the residual deflection of the rail after bending also increases. The simulation results also indicate that with constant top bending support distances, increasing the bending stroke leads to an increase in the bending load. With constant bending strokes, larger support distances require smaller bending loads. When bending loads are constant, larger bending support distances necessitate larger bending strokes. Moreover, as the top die width increases, both the bending stroke and bending load increase.

By changing the width of the top pick and the distance between the supporting picks, the corresponding simulation data are obtained, as shown in Tables 2, 3 and 4.

**Table 2:** Theoretical and simulated stroke of a 100 mm wide top pick.

Support distance	Theoretical calculated stroke	Finite element simulation stroke	Error
600	3.243	3.260	$-0.017$
700	2.398	2.393	0.005
800	1.662	1.671	$-0.009$
900	1.232	1.228	0.004
1000	0.255	0.267	–0.012

**Table 3:** Theoretical and simulated stroke of a 200 mm wide top pick.

Support distance	Theoretical calculated stroke	Finite element simulation stroke	Error
600	4.255	4.266	$-0.011$
700	3.089	3.101	$-0.012$
800	2.022	2.036	$-0.014$
900	1.380	1.392	$-0.012$
1000	0.347	0.341	0.006

**Table 4:** Theoretical and simulated stroke of a 300 mm wide top pick.

Support distance	Theoretical calculated stroke	Finite element simulation stroke	Error
600	5.333	5.329	0.004
700	3.588	3.593	–0.005
800	2.166	2.172	–0.006
900	1.322	1.311	0.011
1000	0.444	0.432	0.012

## Experimental

To verify the top bending model, an experimental verification was conducted on a custom self-made press. The rail model used in the experiment is a 43 kg rail, its material is U71Mn, the specific parameters are as follows: $E=214GPa$, ${{\sigma }_{s}}=650GPa$, its shape and size are ${{H}_{1}}=12$, ${{H}_{2}}=98$, ${{H}_{3}}=30$, ${{B}_{1}}=114$, ${{B}_{2}}=14.5$, and ${{B}_{3}}=70$.

**Figure 24:**
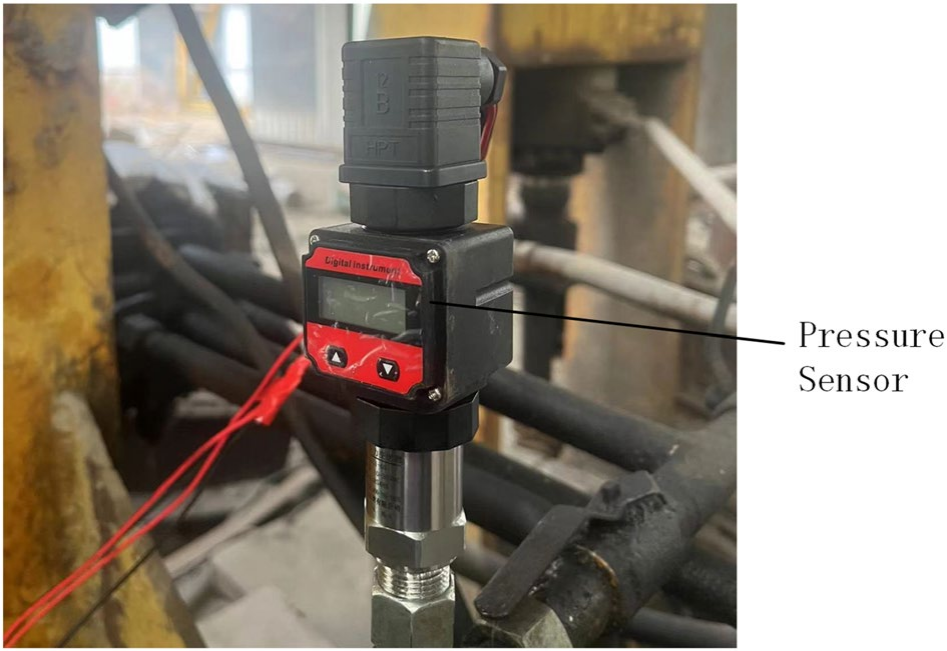
Experimental site.

**Figure 25:**
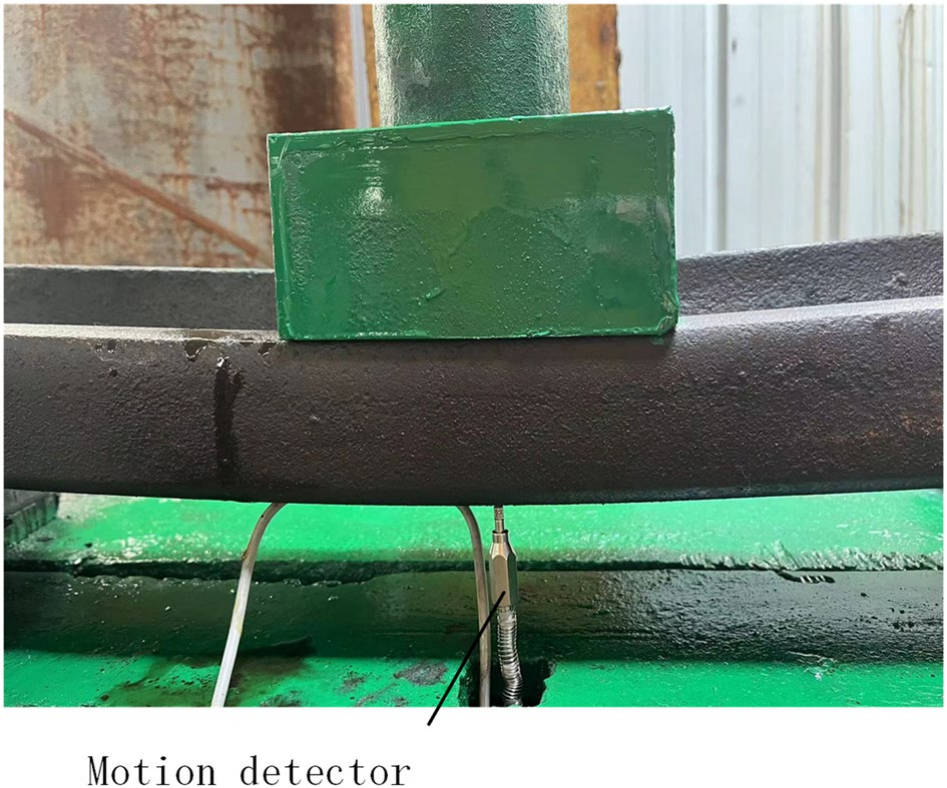
Experimental site.

Figures 24 and  25 show the top bending pressure testing machine. During the top bending process, both ends are supported by support picks. The support distance can be adjusted according to needs. The two picks are symmetrically distributed, and the top pick is located in the middle position. When a load is applied to the rail, the load and deformation produced during the top bending process of the rail need to be measured to verify the correctness of the model. Therefore, this experimental device uses a pressure sensor and a displacement sensor to measure the force and deformation of the rail.

**Figure 26:**
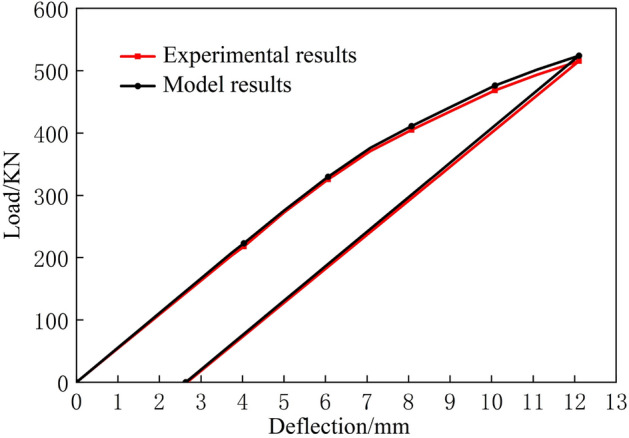
Theoretical calculation and experimental results.

Multiple sets of experiments were conducted on the rail under different top die widths and support die distances, yielding experimental results under various bending conditions. Figure 26 shows a set of comparison diagrams for the experiment. Under the same parameter settings, the slope of the elastic stage in the load-deflection curve is relatively close, the rebound curve in the rebound stage is close to a linear distribution, and its slope *k* is roughly the same as that of the elastic stage; these results verify the feasibility of using simple rebound in the model. By comparing the theoretical calculation curve and the experimental result curve, under the same top bending deflection, the calculated load of the prediction model is higher than the experimental value, and its error increases with increasing deflection. This phenomenon potentially occurs because large size rails have errors due to neglecting the tangential stress. In addition, although the plane assumption is maintained during the bending process of the rail, in the case of large deformation, the neutral plane of the rail will shift; however, this situation not considered in this study. In addition, the material hardening model of the rail may be more complex in the case of larger bends. Due to the increase in strain, the error of the bilinear strengthening model will increase and will no longer be suitable for this model. A more accurate material strengthening model is needed.

**Figure 27:**
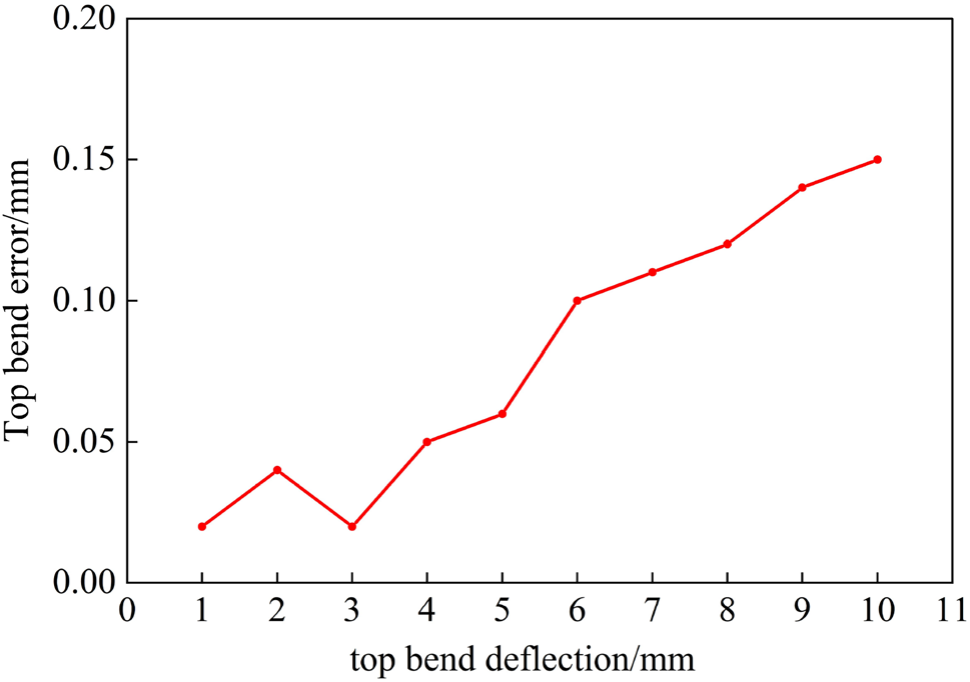
Errors corresponding to different top bend deflections.

Due to space limitations, as shown in Fig. 27, the data for the top bend were obtained using the prediction model. The error between the rail top bending deflection obtained from the experimental results and the target deflection is less than 0.2 mm. Therefore, the top bending model established in this study is reasonable and can provide theoretical support for automatic top bending of the rails.

## Conclusion

In this study, the three-point bending model for a basic rail was optimized by considering the influence of the top die width; this resulted in a corresponding prediction model for basic rail bending. This refinement enhanced the accuracy of the computational results, providing theoretical support for automated bending equipment for basic rails. Analysis of the existing results led to the following conclusions.

1. Theoretical model optimization:
  In the simplification of the mechanical model, the force on the top die was no longer simplified as a concentrated force; rather, two concentrated forces were applied on either side of the top die. Based on the observation, the stress distribution on the top die experienced forces only on its sides, validating the correctness of the mechanical model simplification. Introducing the top die width, which is the distance between the two concentrated forces, successfully yielded the bending moment at different sections of the rail. Building upon elastic-plastic theory, the bending deflection model for top bending was successfully established, and the final optimized prediction model was obtained through curvature relationships. Applying the optimized model to automated bending equipment for basic rails provided more accurate theoretical guidance for achieving automated bending.
2. Confirmation of the rationality and improvement effect of the model via experiments:
  In existing studies of the model, only a fixed top die width is typically considered, and bending is achieved by varying the support die distance. However, in this study, the top die width was varied in both the finite element models and the experiments for observation of its effect on bending. The top die width imposed a limiting effect on the rebound stage. Specifically, a wider top die width led to poorer rebound effects and greater residual deflection after bending. When the influence of the top die width was not considering, a decrease in the support die distance led to increasing errors. The optimized model proposed in this study could mitigate this influence and provide better predictions.
3. Suggestions for further research in complex situations:
  Under large deformation conditions, based on the experimental results, the errors gradually increased. Analysis of the model-building process revealed that the use of a bilinear hardening model for calculating the bending moment introduced errors due to the increase in strain leading to increased stress errors; consequently, this resulted in subsequent calculation inaccuracies. In future research, further refinement of the material hardening model is recommended to improve its accuracy. When conducting elastic-plastic bending analysis on beams with complex cross-sectional shapes, the construction process of the optimized model in this study can be referenced. Adjustments to the corresponding bending moment calculation can then be made to derive the corresponding equations.

## Data Availability

The datasets generated and analysed during the current study are not publicly available due to signing a confidentiality agreement with Party A, but are available from the corresponding author on reasonable request.
